# Simplified State Interaction for Matrix Product State Wave Functions

**DOI:** 10.1021/acs.jctc.1c00674

**Published:** 2021-12-03

**Authors:** Leon Freitag, Alberto Baiardi, Stefan Knecht, Leticia González

**Affiliations:** † Institute for Theoretical Chemistry, Faculty of Chemistry, University of Vienna, Währinger Street 17, 1090 Vienna, Austria; ‡ Laboratory for Physical Chemistry, ETH Zurich, Vladimir-Prelog-Weg 2, 8093 Zurich, Switzerland; § GSI Helmholtz Centre for Heavy Ion Research, Planckstr. 1, 64291 Darmstadt, Germany

## Abstract

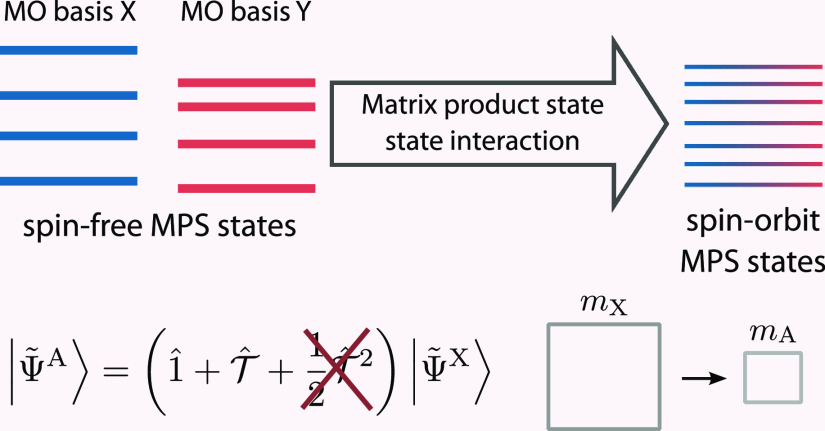

We present an approximation to the state-interaction approach for matrix product state (MPS) wave functions (MPSSI) in a nonorthogonal molecular orbital basis, first presented by Knecht et al. [*J. Chem. Theory Comput.,***2016**, *28,* 5881], that allows for a significant reduction of the computational cost without significantly compromising its accuracy. The approximation is well-suited if the molecular orbital basis is close to orthogonality, and its reliability may be estimated a priori with a single numerical parameter. For an example of a platinum azide complex, our approximation offers up to 63-fold reduction in computational time compared to the original method for wave function overlaps and spin–orbit couplings, while still maintaining numerical accuracy.

## 1 Introduction

Accurate calculations of many photochemical processes can be a daunting task. Excited states are often governed by strong electron correlation effects and many close-lying excited states, where multiconfigurational electronic structure methods^1,2^ are indispensable.

Multiconfigurational methods based on the complete active space self-consistent field (CASSCF)^3^ are well-established for handling strong correlation effects. These approaches require selecting an orbital subspace called *active orbital space* whose size determines the computational cost. Traditional CASSCF methods scale exponentially with the number of the active orbitals and electrons, allowing for calculations of up to 22 electrons in 22 orbitals with a massively parallel approach^4^ but limiting its size to approximately 18 electrons in 18 orbitals^5^ under more moderate computational time requirements. These limits can be reached very quickly, especially in polynuclear transition-metal complexes. One approach to overcome the exponential scaling of CASSCF is the density matrix renormalization group (DMRG)^6,7^ for quantum chemistry,^8−16^ which, combined with self-consistent field orbital optimization (DMRG-SCF),^17,18^ is able to variationally approximate CASSCF wave functions to arbitrary accuracy at a polynomial instead of exponential scaling of the computational cost.

In the CASSCF paradigm, and also with DMRG-SCF, excited states are usually calculated with a *state-average* ansatz, where a single orthonormal set of molecular orbitals (MOs) is optimized to provide a balanced representation of several states. This allows for a straightforward calculation of transition densities and moments that are required to compute properties such as oscillator strengths, magnetic properties, or spin–orbit couplings. However, state-averaging is not always possible or desired: (i) the individual state characters differ too much for an average set of orbitals to yield an adequate description; (ii) state-averaging, for example, between different spin multiplicities, is not supported by the computer implementation of the method, or (iii) a single molecular set of orbitals is simply not possible at all. The latter problem is encountered, for instance, when calculating the overlap between wave functions that are associated with different molecular structures to monitor the change in the character of the electronic wave function, as described in ref (19). In such cases, each state is optimized independently and the resulting MO bases for the individual states are no longer the same. As a consequence, the states are no longer mutually orthogonal, turning the calculation of transition densities and moments into a challenging task.

A solution to this predicament is to use the *complete active space state interaction* (CASSI) method, proposed by Malmqvist and Roos,^20,21^ who suggested to transform the MO bases for the individual states to a biorthonormal basis. Along with the orbital rotation, this requires a simultaneous “counter-rotation” of the wave function expansion coefficients: for a configuration interaction (CI)-type wave functions, which include CASSCF wave functions, this step can be achieved with a series of single-orbital transformations.^21,22^ After transformation to biorthonormal basis, wave function overlaps and transition densities may be evaluated at little to no computational overhead.

The CASSI approach was soon extended to calculate spin–orbit couplings,^23^ and with the advent of DMRG for quantum chemistry, the DMRG-based version of CASSI, later named the *matrix product state* (MPS) *state interaction* (sic!) (MPSSI), has been introduced.^24^ To account for spin–orbit interaction with DMRG wave functions, several other approaches based on spin-free wave functions that share a *common* MO basis were developed^25−27^ as well as a fully relativistic four-component approach.^28,29^

Multiconfigurational methods and, specifically, the CASSCF method are often used as an underlying method for the electronic structure calculations of ab initio nonadiabatic dynamics:^30^ partially due to their computational efficiency for small systems and ability to describe strong correlation but also because of the readily available implementations for gradients and nonadiabatic couplings.^31−34^ With the help of the CASSI method, ab initio nonadiabatic excited-state dynamics with spin–orbit couplings, for example, with the SHARC approach,^35^ may be employed to study processes involving different spin states coupled via intersystem crossing. Additionally, overlaps between wave functions at different time steps may also be calculated with CASSI and may serve to approximate nonadiabatic couplings that are included in the on-the-fly propagation of nuclear wave functions.^36^ The steep scaling of CASSCF with respect to the active orbital space may be tamed with DMRG-SCF also with surface-hopping dynamics, as the calculation of the analytical gradients and nonadiabatic couplings has been recently reported,^37^ and spin–orbit couplings and wave function overlaps may be calculated with MPSSI. DMRG-SCF, despite its polynomial scaling of the computational time with the active space size, is, nevertheless, computationally very intensive, and MPSSI is also a cost-intensive method with a computational cost comparable to that of DMRG-SCF. Dynamics calculations, however, require a cheap and performant electronic structure method, as electronic structure calculations of energies, gradients, and couplings are carried out for hundreds or thousands of time steps. Accordingly, elimination of every possible bottleneck in the electronic structure calculations is extremely beneficial for dynamics calculations.

With the aim of making DMRG broadly applicable to ab initio molecular dynamics simulations, in the present work, we identify the main computational bottlenecks of an MPSSI calculation. Then, we extensively benchmark the sensitivity of the MPSSI accuracy to the choice of the simulation parameters and identify the simulation setup that yields the best compromise between computational cost and accuracy. This optimal setup relies on two approximations, that is, a simple-yet-effective implementation of the orbital rotation operator and an efficient MPS truncation scheme. The error introduced in these two steps is controlled by a single parameter that can, therefore, be tuned based on the target simulation accuracy. We demonstrate the effectiveness of these approximations by means of MPSSI calculations of wave function overlaps and spin–orbit couplings for a medium-sized transition-metal complex.

## 2 Theory

As the starting points of this work, we first outline the CASSI and the MPSSI approaches. We assume two sets of multiconfigurational wave functions |Ψ^X^⟩ and |Ψ^Y^⟩, each expressed in their own MO basis {ϕ_*p*_^X^} and {ϕ_*p*_^Y^}, respectively, which are not mutually orthogonal. The goal of the CASSI approach is to find the biorthonormal MO bases {ϕ_*p*_^A^} and {ϕ_*p*_^B^} such that
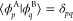
1and the corresponding transformation of the wave functions |Ψ^X^⟩ and |Ψ^Y^⟩ such that the transition matrix elements  of any operator  may be calculated with very little additional computational effort compared to the case where |Ψ^X^⟩ and |Ψ^Y^⟩ belong to the same MO basis. To this end,^21^ the LU decomposition of the inverse of the orbital overlap matrix **S**^XY^ (with *S*_*pq*_^XY^ = ⟨ϕ_*p*_^X^|ϕ_*q*_^Y^⟩) is constructed
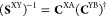
2

The **C**^XA^ and **C**^YB^ matrices define the transformation from the MO to the biorthogonal basis such that
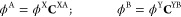
3

Before proceeding to the transformation of the wave functions |Ψ^X^⟩, let us briefly introduce the wave function ansatz employed with DMRG, the MPS. A general CI ansatz for an arbitrary wave function |Ψ⟩ in a Hilbert space spanned by *L* spatial orbitals may be expressed as
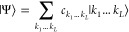
4with *c*_*k*_1_...*k*_L__ as the CI coefficients and |*k*_1_...*k*_*L*_⟩ as occupation number vectors. The notation |*k*_1_...*k*_*L*_⟩ reflects the fact that for each spatial orbital *l*, we may have a local basis state |*k*_*l*_⟩ = {|↑↓⟩, |↑⟩, |↓⟩, |0⟩} and the total occupation number vector consists of local occupations of all orbitals 1, ..., *L*.

The CI coefficients *c*_*k*_1_...*k*_L__ may be reshaped as an *L*-dimensional tensor and decomposed^38,39^ by repeated application of the singular value decomposition into a product of matrices **M**^*k*_*l*_^, yielding an MPS
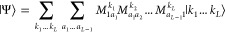
5

The dimension of matrices (i. e., the *a* indices) may be limited to a certain maximum dimension *m*, usually referred to as the *number of renormalized block states* or *maximum bond dimension*. This way, the number of parameters entering the wave function *ansatz* definition is reduced from exponential, as it is in full CI, to polynomial. The optimization of MPS wave functions is most commonly carried out with the DMRG approach, for the explanation of which we refer the reader to the comprehensive reviews of Schollwöck^38,39^ and ref (16).

Analogously to the MPS, operators may be expressed in a *matrix product operator* (MPO) form as
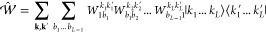
6

We consider next the transformation algorithm for wave functions |Ψ^X^⟩, when |Ψ^X^⟩ are MPSs, as introduced in ref (24). We perform another LU decomposition, this time of the **C**^XA^ matrix, and from its lower and upper triangular parts (**C**_L_^XA^ and **C**_U_^XA^, respectively), we construct the matrix **t**, with its lower and upper triangular part being
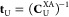
7
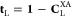
8

The matrix **t** is then used to transform the wave functions |Ψ^X^⟩ as follows

- First, the inactive orbitals are transformed by scaling the MPS with a factor α given by
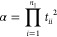
9where *i* runs over all inactive orbitals.

- For the subsequent transformation with respect to the *active* orbitals, the following steps are repeated for each active orbital *l*:
  i Each matrix **M**^*k*_*l*_^ is multiplied with *t*_*ll*_^2^ for *k*_*l*_ = |↑↓⟩ and with *t*_*ll*_ for *k*_*l*_ = |↑⟩ and |↓⟩,
  ii an MPO  is applied to the scaled MPS |Ψ̃^X^⟩ yielding a transformed MPS
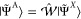
10with
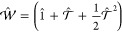
11and
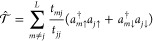
12
  iii In the last step, one performs an SVD compression of |Ψ̃^A^⟩ to obtain the final MPS |Ψ^A^⟩, a representation of the original state in the biorthonormal basis {ϕ^A^}. Analogously, |Ψ^B^⟩ may be constructed by repeating the steps mentioned above with the **C**^YB^ matrix and |Ψ^Y^⟩ MPS. |Ψ^A^⟩ and |Ψ^B^⟩ are then employed to calculate transition density matrix elements and properties.

While the original MPS transformation algorithm has been shown to be highly accurate for various properties, including spin–orbit couplings and g-factors of actinides,^24^ its current implementation has two major bottlenecks.

The first bottleneck originates from the SVD compression from step (3): the application of the MPO in eq 10 to an MPS results in a transformed MPS with the maximum bond dimension of *b* × *m*, where *b* is the maximum bond dimension of the MPO  and *m* is the maximum bond dimension of the MPS |Ψ̃^X^⟩. The final SVD compression in step (3) reduces the final bond dimension of the transformed MPS, which is necessary since the storage size of the MPS and the cost of transition density matrix element evaluation^40^ scales with  and thus becomes prohibitively expensive for large *m*. However, MPS compression itself is a computationally expensive step with a computational cost of  and constitutes a crucial bottleneck in MPSSI. The original MPSSI implementation^24^ employs a fixed value of *m* = 8000, preserving the expectation value of the energy up to 10^–8^ a. u., but at a price of significant computational cost.

The second bottleneck arises from the construction of the MPO : as shown in eq 11, this step requires the construction of the  operator, which is not trivial. The original implementation^24^ avoids this problem by calculating  and  and adding the resulting MPS afterward. Applying the  operator twice, however, increases the bond dimension of the resulting MPS by a factor of *b*^2^ and requires an additional MPS compression step between the first and the second application of .

In this work, we improve the efficiency of the MPSSI method by introducing two simple but effective changes to the MPS transformation algorithm. The first is the first-order approximation of eq 11 by neglecting the final second-order term. This approximation may be justified as follows: since to allow for the formation of biorthogonal bases, the original MO bases have to be sufficiently similar, i. e., show an overlap fairly close to unity, the resulting ***t*** matrix should not deviate significantly from the identity matrix. Equation 11 can be thought of as being a second-order Taylor approximation to the exponential of , and in the regime of ***t*** close to identity also, a linear approximation should hold. While accounting through the application of  for a full rotation of a singly occupied orbital *j* in a given many-particle basis state of the complete many-particle wave function, neglecting the  term corresponds to an approximation of the full effect of the rotation for a corresponding doubly occupied orbital *k*. In general, the latter requires the application of a two-electron excitation operator *e*_*pkqk*_ = *E*_*pk*_*E*_*qk*_ – δ_*kq*_*E*_*pk*_.^21^ Hence, neglecting , as proposed in the present work, corresponds to approximating the two-electron excitation operator *e*_*pkqk*_ for the transformation of a doubly occupied orbital *k* in a given many-particle basis state by a sum of one-electron excitation operators, that is, *e*_*pkqk*_ ≈ *E*_*qk*_ – δ_*kq*_*E*_*pk*_. Furthermore, eq 12 shows that the  operator is scaled with the ratio between the off-diagonal and diagonal elements of . For an orthogonal basis,  will be the identity matrix and this ratio will be zero. Therefore, a simple estimate based on off-diagonal elements of ***t***, such as the *L*^2^ norm of ***t*** – ***I***_***L***_ (with ***I***_***L***_ as an *L* × *L* identity matrix), may be employed as a measure of the accuracy of the approximation.

The second approximation is the reduction of the maximum bond dimension of the compressed MPS, therefore reducing the computational cost of the MPS compression. We highlight that the speedup associated with the reduction of the maximum bond dimension employed in the MPS compression step comes at the price of losing accuracy in the approximation of the full-CI wave function as an MPS. Specifically, the error associated with this truncation step will increase with the difference between the two sets of nonorthogonal molecular orbitals, as will be demonstrated later in the results. However, two sets of molecular orbitals that are obtained for two different spin configurations and based on the same molecular structure are often not drastically different. This is the reason why, as we will show in the following, the MPS can be largely compressed without compromising the accuracy of the matrix elements of the spin–orbit coupling operator. The compression scheme may become less efficient in more complex cases, such as for calculating transition properties between orbitals obtained for different excited states calculated at different nuclear geometries. Still, it would be possible in these cases to adapt the bond dimension *m* to yield a given target wave function accuracy that is selected a priori, as discussed in ref (41). Alternatively, the loss of accuracy consequent to the MPS compression can be monitored by calculating the expectation value of operators that are associated with conserved quantum numbers before and after the truncation. As we showed in our original work on MPSSI, a small change in the squared spin operator indicates that the wave function accuracy is preserved after the truncation step.

In the following section, we demonstrate that both approaches significantly improve the computational cost of MPSSI with almost no effect on accuracy. Although both steps reduce the accuracy of the transformation, the following numerical test demonstrate that the errors introduced are negligible for several types of properties.

## 3 Numerical Examples

As a testbed we employ trans, trans, trans-[Pt(N_3_)_2_(OH)_2_(NH_3_)_2_] (in the following referred to as **1**), which is a flagship Pt(IV) azide complex, relevant in photoactivated cancer chemotherapy.^42−44^ As the majority of 5d metal compounds, **1** shows strong spin–orbit couplings and since its photoactivation mechanism involves azide dissociation, such a process is best described by multiconfigurational methods.^45^

### 3.1 Performance of MPSSI Approximation on Wave Function Overlaps

In principle, CASSI/MPSSI allows for an easy calculation of wave function overlaps constructed with nonorthogonal orbital sets. Wave function overlaps, especially between states at different molecular structures or spin multiplicities, are widely used in ab initio excited-state molecular dynamics^19,36,46,47^ or in wave function analysis.^48^ Here, we investigated the accuracy of the linear approximation to  (in the following called “MPSSI approximation”) for wave function overlaps of both ground and excited states for varying molecular structures of the same molecule. With an increasing deviation of molecular structures, the dissimilarity of the orbitals and the *L*^2^ norm of the ***t*** – ***I***_***L***_ matrix also increases, allowing us to also assess the limits of the MPSSI approximation with the increasing norm.

We performed CASSCF and DMRG-SCF calculations with a comparably small active space of eight electrons in nine orbitals. This active space is capable to qualitatively describe the energies of the lowest excited states and is also small enough for DMRG-SCF to be able to reproduce the CASSCF results almost exactly: the final DMRG-SCF energies differ from their CASSCF counterparts by no more than 10^–7^ a. u.

**Figure 1:**
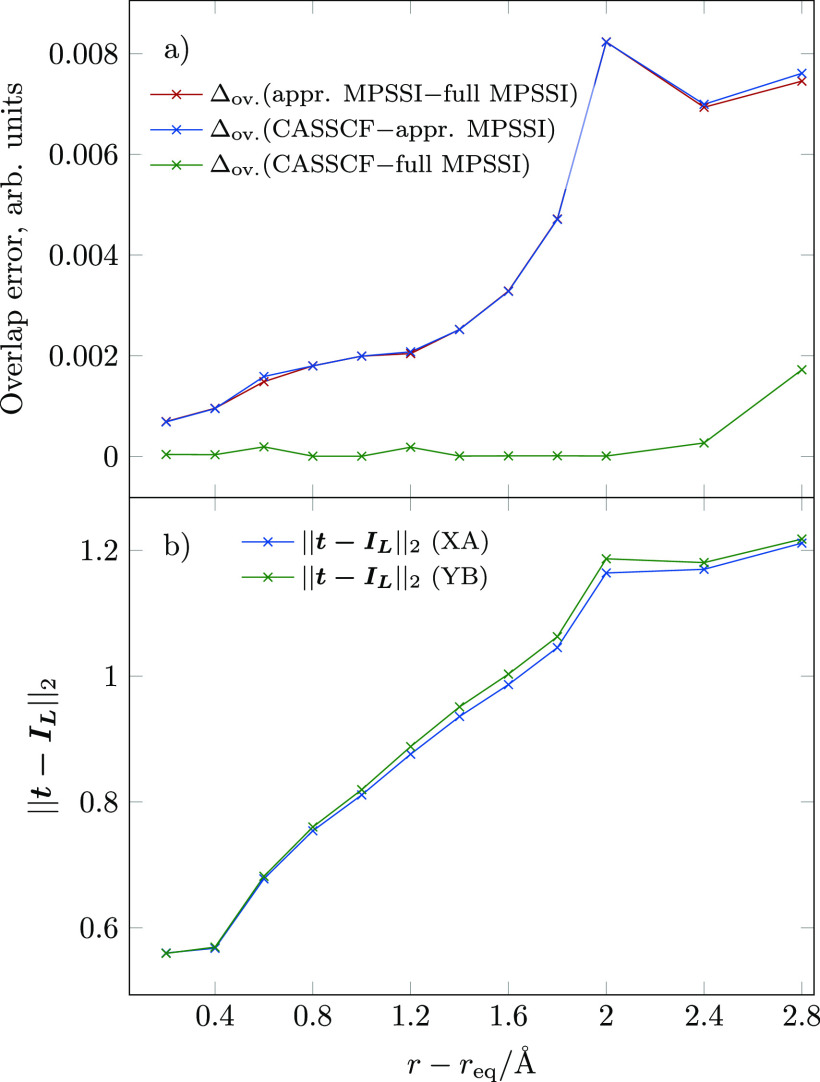
(a) Average differences of overlaps calculated with CASSCF and full and approximate MPSSI; (b) *L*^2^ norm of ***t*** – ***I*_*L*_** matrices, with ***I*_*L*_** as the identity matrix; XA corresponds to the orbitals at the equilibrium structure and YB to orbitals at a given *r* – *r*_eq_.

We performed a rigid scan along the Pt–N bond of one of the azide ligands with CASSCF and DMRG-SCF and calculated the wave function overlap of the lowest five singlet states at structures with an elongated Pt–N bond with their counterparts at the equilibrium structure. We calculated the overlaps of the CASSCF wave functions with CASSI and those of DMRG-SCF wave functions with full and approximate MPSSI: the average pairwise differences between these are shown in Figure 1a. The overlap difference between CASSCF and full MPSSI (green line) reflects the error arising only due to DMRG approximation to the CASSCF wave function. The effect arising due to the MPSSI approximation can be fully estimated from the approximate to full MPSSI difference (red line). The corresponding changes in the *L*^2^ norm of ***t*** – ***I*_*L*_** matrices are shown in Figure 1b.

Given the tightly converged DMRG-SCF wave function, the errors arising due to the DMRG approximation are negligible: for all *r* – *r*_eq_ values except 2.8 Å, the overlap error is less than 3 × 10^–4^, whereas for the latter calculation, it rises slightly to 2 × 10^–3^. This discrepancy is due to a slightly poorer convergence of the wave function at this particular *r* – *r*_eq_ value than for other Pt–N bond lengths. Recalling that in contrast to the quadratic convergence of the energy, property calculations converge linearly with respect to the wave function quality, the maximum energy error for this case is closer to 10^–7^ a. u., whereas for other Pt–N bond lengths, it is well below this value. Nevertheless, all of these errors are so small that they may be considered negligible. The MPSSI approximation error is, however, larger than the DMRG approximation error for all calculations and rises with increasing ***t*** norm: starting with approximately 4 × 10^–4^ at *r* – *r*_eq_ = 0.2 Å with a corresponding ***t*** – ***I*_*L*_** norm of 0.4 (and thus remaining in the same order of magnitude as the DMRG approximation errors), it steadily increases with increasing ***t*** – ***I*_*L*_** norm, reaching values of 8 × 10^–3^ for the extended Pt–N bonds.

In the range of *r* – *r*_eq_ of 1.2 to 1.8 Å, we see a particularly large increase in the MPSSI approximation error, which corresponds to ***t*** – ***I*_*L*_** norm values between 0.9 and 1. Therefore, we propose a conservative cutoff ***t*** – ***I*_*L*_** norm value of 1, below which we recommend to use the approximation. This choice is, however, largely arbitrary: the average overlap error at the cutoff value is 2 × 10^–3^, and even the largest error value of 8 × 10^–3^ in these calculation series is still sufficient for a qualitatively correct calculation.

**Figure 2:**
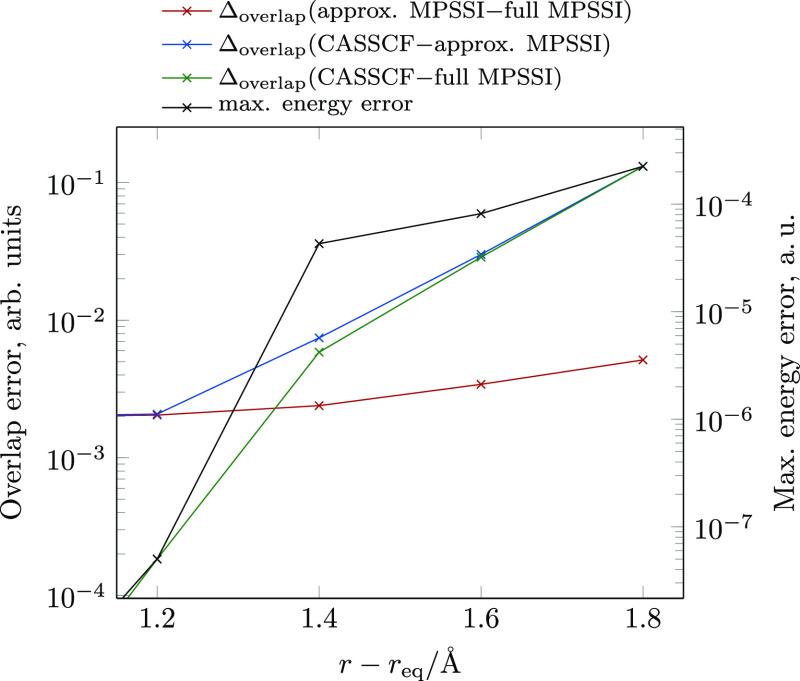
Average overlap error for overlaps with CASSCF and full and approximate MPSSI, for a partially converged DMRG-SCF wave function. Maximum energy error with respect to a corresponding CASSCF calculation is shown in black.

The suitability of larger MPSSI approximation errors for qualitative calculations is best illustrated if one compares the results to those from a partially converged DMRG-SCF calculation, which is a common practice in the literature. Figure 2 shows the same overlap errors displayed in Figure 1a but for partially converged DMRG-SCF wave functions, where energy differences to the corresponding CASSCF wave function are up to 2 × 10^–4^ a. u. The norms of the ***t*** – ***I*_*L*_** matrices are similar and the MPSSI approximation errors are almost the same as the corresponding errors for the fully converged DMRG-SCF wave functions. However, the errors arising due to the DMRG approximation increase sharply with the decreasing DMRG-SCF wave function quality. For energy errors in the range of 10^–4^ a. u. to 10^–5^ a. u., typical for large-scale DMRG calculations, the order of magnitude of the MPSSI approximation and the DMRG approximation error is similar, and therefore, approximate MPSSI is still suitable for qualitative calculations.

We may conclude that the MPSSI approximation error is independent of the DMRG wave function quality but rather depends only on the ***t*** matrix. Thus, the *L*^2^ norm of the ***t*** – ***I*_*L*_** matrix constitutes an easy metric available prior to the MPSSI rotation that allows a simple decision whether the MPSSI approximation should be employed or not.

### 3.2 Performance of MPSSI Approximation on Spin–Orbit Couplings

Here, we investigate the MPSSI approximation performance in the calculation of spin–orbit coupling matrix elements, which is another typical use case for MPSSI. We employ the same active space of eight electrons in nine orbitals as in the previous example but calculate energies and spin–orbit coupling matrix elements for the five lowest singlets and triplet states of **1** at the equilibrium structure.

**Figure 3:**
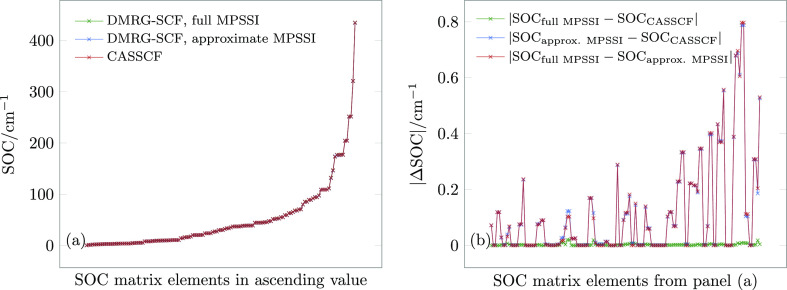
(a) Spin–orbit coupling (SOC) matrix elements for the first five singlet and five triplet states at the equilibrium structure of **1** for an active space of eight electrons in nine orbitals. (b) Absolute errors of the SOC matrix elements in panel (a).

Figure 3a shows spin–orbit couplings calculated with CASSCF and DMRG-SCF employing the original (full)^24^ and approximate MPSSI scheme. As in the previous example, DMRG-SCF wave functions have been converged so that the DMRG-SCF energies differ from their CASSCF counterparts by less than 10^–7^ a. u.: therefore, any error arising from the DMRG approximation is negligible. The differences between the calculated values are displayed in Figure 3b and show that the effect of the DMRG approximation (green curve) is indeed negligible: the largest error due to the DMRG approximation does not exceed 0.02 cm^–1^. The error due to the MPSSI approximation is, similarly to the previous example, slightly larger but still negligible for all practical purposes: the average error is 0.077 cm^–1^ and the maximum error is approximately 0.8 cm^–1^.

**Figure 4:**
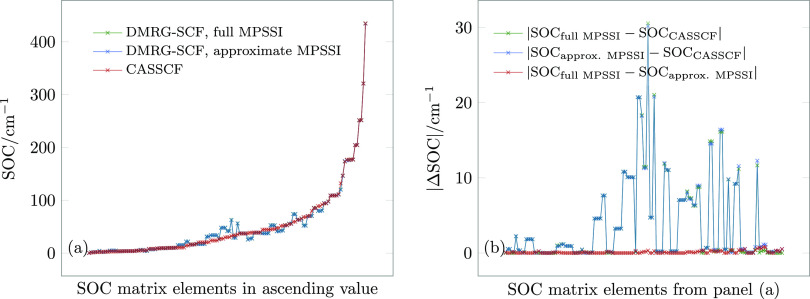
(a) Spin–orbit coupling (SOC) matrix elements for the first five singlet and five triplet states at the equilibrium structure of **1** with a partially converged DMRG-SCF wave function. (b) Absolute errors of the SOC matrix elements in panel (a).

As in the previous example, we also consider the case of a partially converged DMRG-SCF wave function, where the energies of some states differ up to 10^–5^ a. u. from their CASSCF counterparts. This accuracy is typical for large-scale DMRG-SCF calculations and is more than sufficient for accurate absorption energies up to 10^–5^ a. u. The results are displayed in Figure 4. We note that in this case, the SOC error arising from the DMRG-SCF approximation increases by several orders of magnitude up to 30 cm^–1^, while the MPSSI approximation error remains the same. Thus, in this case, the total error in the DMRG calculation largely consists of the DMRG approximation error, while the MPSSI approximation error is completely negligible.

**Figure 5:**
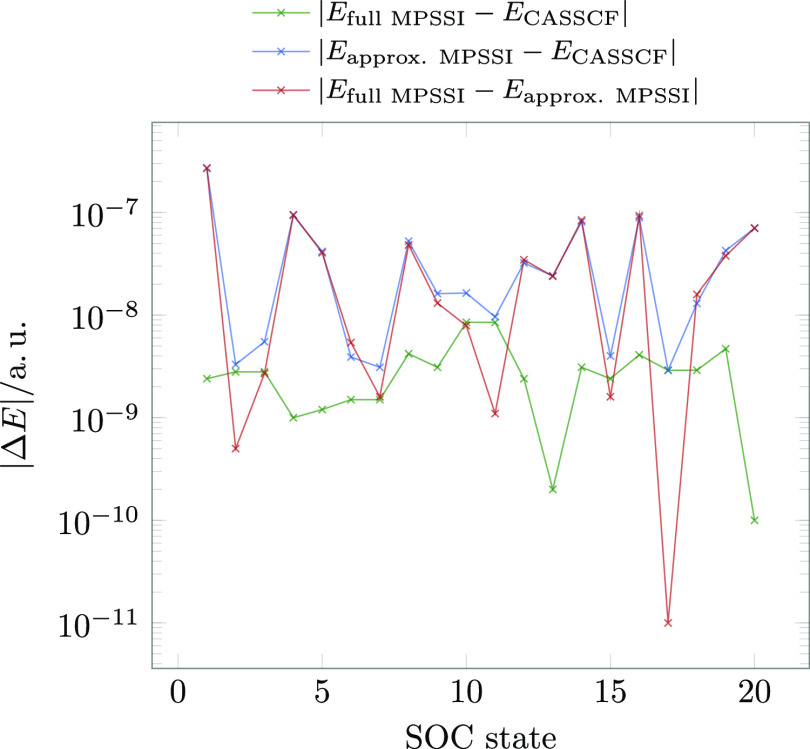
Errors of spin–orbit corrected energies calculated with CASSI and full and approximate MPSSI at the equilibrium structure of **1** for the first 20 spin–orbit coupled states arising from spin–orbit coupling of five singlets and five triplets. The active space employed in this calculation consisted of eight electrons in nine orbitals.

Figure 5 shows the absolute errors of spin–orbit corrected energies. All errors remain below 10^–6^ a. u. and thus negligible.

Finally, we would like to mention the computational time savings arising from the approximation. Due to the small size of the active space, the MPSSI approximation is not the bottleneck in this calculation, but it already reduces the computational time by approximately 40%, that is, from 10 min 7 s to 6 min 6 s of run time on 4 cores of an Intel Xeon E5-2650 CPU.

### 3.3 Performance of the Methods with a Larger Active Space

From a calculation using time-dependent density functional theory (TD-DFT, CAM-B3LYP/def2-TZVPP) and including several low-lying singlet excited states of **1**, we know that CASSCF and DMRG-SCF calculations with an active space of eight electrons in nine orbitals, as employed in the previous section, cannot even qualitatively account for the spin–orbit couplings: the largest absolute value for the spin–orbit coupling between the five lowest singlet and triplet excited states was 434 cm^–1^, whereas the corresponding value from a TD-DFT calculation was found to be approximately 1800 cm^–1^.

**Figure 6:**
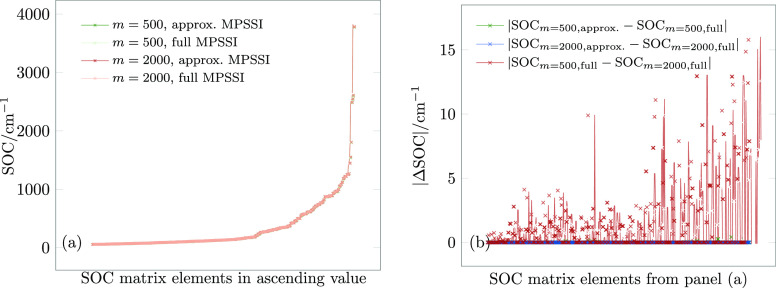
(a) Spin–orbit coupling (SOC) matrix elements for the first 10 singlet and 9 triplet states at the equilibrium structure of **1**, for an active space of 26 electrons in 19 orbitals. (b) Absolute errors of the SOC matrix elements in panel (a).

This insufficiency can be remedied by a DMRG-SCF calculation with 26 electrons in 19 orbitals, as employed in ref (45). As this active space is computationally too expensive for a CASSCF calculation, only DMRG-SCF calculations with subsequent approximate and full MPSSI calculations are performed. In addition, we assess the error arising due to the MPS compression step in the MPS transformation by testing various *m* values for the compressed MPS: the DMRG-SCF calculations were performed for *m* = 500, but during the MPSSI procedure, the intermediate MPS during rotation was compressed either to the original *m* = 500 or to *m* = 2000. Note that we could not afford a postcompression *m* value of 8000 from the original paper of Knecht et al.^24^ due to its prohibitive computational requirements. Furthermore, in the following, we consider the 10 lowest singlet and 9 triplet states. The calculated SOC and their errors are shown in Figure 6.

As can be seen from Figure 6a, all methods yield almost identical values for the SOC. A closer look at errors (Figure 6b) reveals a maximum error of approximately 15 cm^–1^, which arises entirely due to the MPS compression. The MPSSI approximation error is negligible: the maximum MPSSI approximation error is 0.41 cm^–1^ for *m* = 500 and just 1 × 10^–3^ cm^–1^ for *m* = 2000. The small MPSSI approximation error is not surprising for this calculation, as the ***t*** – ***I*_*L*_** norms are only 0.002 and 0.006. We can also see that the MPSSI approximation is affected by compression but only very slightly: it is the compression error in the first place that contributes to the total error, which is nevertheless still small enough for quantitative results.

**Figure 7:**
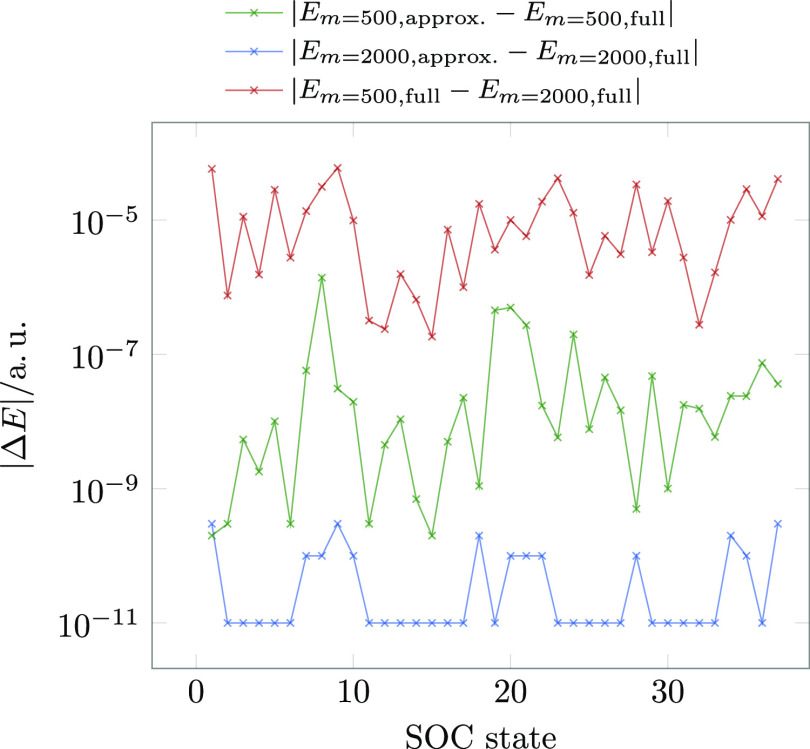
Errors of spin–orbit corrected energies calculated with full and approximate MPSSI for *m* = 500 and *m* = 2000 at the equilibrium structure of **1** for the first 37 spin–orbit coupled states. The active space employed in this calculation consisted of 26 electrons in 19 orbitals.

The errors in the spin–orbit corrected energies are analyzed in Figure 7. Also, here, the largest error in energies arises solely due to the compression. With an average error of 1.3 × 10^–5^ a. u. or 3 × 10^–4^ eV, it is also almost negligible. The MPSSI approximation error alone for both *m* = 500 and 2000 are at least 2 orders of magnitude smaller and are at the same order of magnitude as typical convergence thresholds for the SCF procedure and way lower than the expected DMRG truncation error: it can be safely neglected. It is noteworthy, however, that the MPSSI approximation error increases slightly for the lower-quality *m* = 500 DMRG wave function, implying a small direct effect of the compression on the approximation.

Table 1 discloses the massive speedup that both the MPSSI approximation and the compression entail. Compared to a full MPSSI calculation with *m* = 2000, the MPSSI approximation gains a 7.5-fold speedup, the compression alone to *m* = 500 gains a 18-fold speedup, and the combination of both methods gains an overwhelming 63-fold speedup. Both, the MPSSI approximation and compression are essentially able to eliminate the bottlenecks in the MPSSI method, while still retaining for quantitative accuracy.

**Table 1:** Runtimes for Approximate and Full MPSSI Calculations with Intermediate and Final MPS Compression to *m* = 500 and 2000, as Run on 24 Cores of an AMD EPYC 7502 CPU

*m*	approx.	Full
500	6 h 2 m	21 h 46 m
2000	2 d 3 h	15 d 19 h

Although the smallest *m* value of 500 chosen by us is dictated by the original *m* value employed during the wave function optimization, it is tempting to use an even smaller value to save further computational time. However, given the comparably larger compression error that would increase even further for smaller *m* values, we do not recommend such a reduction.

## 4 Conclusions

In this work, we presented two modifications to the original formulation of the MPSSI method by Knecht et al.,^24^ which despite being simple allow for drastic computational savings while retaining controlled accuracy in the DMRG-SCF calculation of properties.

The first modification, named the “MPSSI approximation”, is based on the omission of the quadratic term in the operator that is employed to counterrotate the MPS, to match the effect of the basis transformation. The second modification consists of decreasing the maximum bond dimension of the intermediate and the final counterrotated MPS by the SVD compression with a smaller *m* value. The accuracy of both modifications may be controlled independently of each other by a numerical parameter. In the case of the MPSSI approximation, it is the *L*^2^ norm of the ***t*** – ***I*_*L*_** matrix employed for the orbital rotation, which is known before the time-consuming MPS counterrotation, and thus allows for an error estimate of the MPSSI approximation beforehand. For the MPS compression, it is the *m* value of the intermediate and the final compressed MPS.

We have tested both modifications in two common useful scenarios where efficiency is highly desired: the calculation of wave function overlaps and spin–orbit couplings. Both quantities are, for example, indispensable to perform efficient ab initio nonadiabatic simulations on the fly. In all our examples, the discrepancies in these properties due to the MPSSI approximation error were very small. For tightly converged DMRG-SCF wave functions, close enough to CASSCF wave functions, the MPSSI approximation error was found to be larger than the DMRG approximation error but unlike the latter not dependent on the wave function quality. Instead, it shows monotonous dependence on the *L*^2^ norm of the ***t*** – ***I*_*L*_** matrix. When DMRG-SCF employs large active spaces, the MPS compression to the original *m* value of the unrotated MPS allows for very substantial computational time savings but introduces an additional source of error: although the MPS compression error is larger than that of the MPSSI approximation, it is still small enough to allow quantitative computation of properties.

In the current calculations, the MPSSI approximation and the MPS compression to *m* = 500 gave us a total 63-fold speedup compared to a calculation with compression to *m* = 2000, while maintaining a total error still small enough for quantitative computation of properties. The compression to *m* = 8000 as in the original implementation could not be performed due to excessive computational requirements: the performance gain compared to such a calculation would have been even larger. We believe that the speedups achieved with the improvements in this work will pave the way to faster and more affordable large-scale multiconfigurational calculations, as well as allow DMRG-SCF to be applied in computationally intensive scenarios, for example, in ab-initio excited-state molecular dynamic simulations.

## Supporting Information Available

Computational details and details regarding the active space orbitals (PDF)
