# Supplementary material for: Simplified State Interaction for Matrix Product State Wave Functions
Source: J Chem Theory Comput. 2021 Dec 3;17(12):7477–85. doi: 10.1021/acs.jctc.1c00674 (PMC8675135; doi:10.1021/acs.jctc.1c00674)
Supplement: Supplementary file 1 — ct1c00674_si_001.pdf [file ct1c00674_si_001.pdf]

Supporting Information: A Simplified State Interaction for Matrix Product State Wave Functions

Leon Freitag,*,† Alberto Baiardi,‡ Stefan Knecht,¶ and Leticia González*,†

†*Institute for Theoretical Chemistry, Faculty of Chemistry, University of Vienna, Währinger Str. 17, 1090 Vienna, Austria*

‡*ETH Zurich, Laboratory for Physical Chemistry, Vladimir-Prelog-Weg 2, 8093 Zurich, Switzerland*

¶*GSI Helmholtz Centre for Heavy Ion Research, Planckstr. 1, 64291 Darmstadt, Germany*

E-mail: leon.freitag@univie.ac.at; leticia.gonzalez@univie.ac.at

Computational Details

The equilibrium gas-phase molecular structure of complex **1** has been taken from Ref. S1. For the overlap rigid scans, the structure was fixed and one Pt–N_3 bond was stretched by up to 2.8 Å from its equilibrium bond length (2.077 Å).

The CASSCF and DMRG-SCF calculations employed the all-electron ANO-RCC valence quadruple-zeta polarised (ANO-RCC-VQZP) basis set[S2] for Pt and its triple-zeta analogon, ANO-RCC-VTZP, for other atoms. Two-electron integrals were calculated with the atomic compact Cholesky decomposition (CD) approach[S3–S5] with a decomposition threshold of 10^{-4} a. u., and the second-order scalar-relativistic Douglas–Kroll–Hess one-electron Hamiltonian[S6–S8] was employed for scalar relativistic effects.

CASSCF and DMRG-SCF calculations were performed with active spaces of 8 electrons in 9 orbitals and 26 electrons in 19 orbitals, shown in Fig. S1.

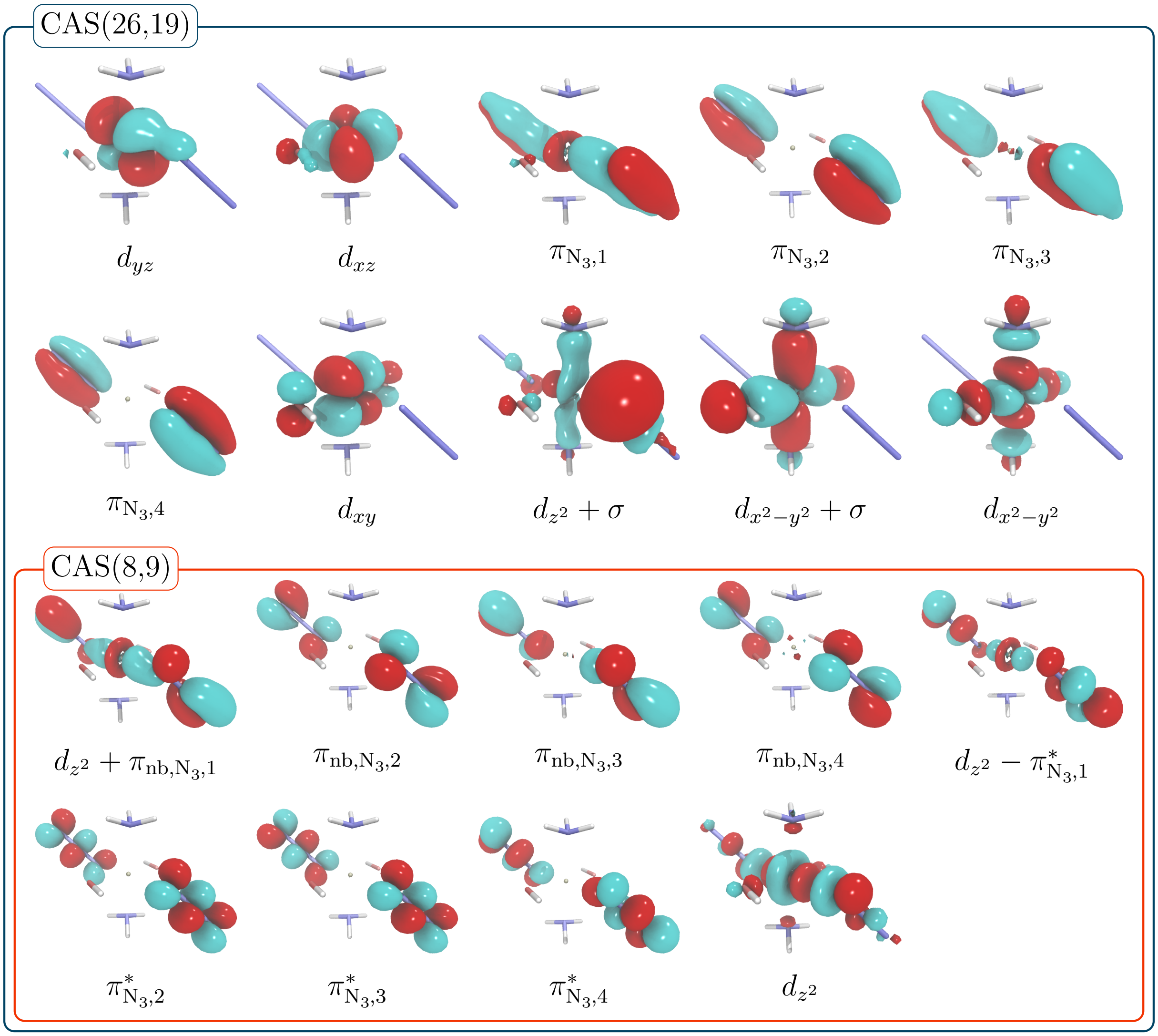

Figure S1: Active orbital spaces employed for CASSCF and DMRG-SCF calculations. The plus or minus signs in the orbital description denote bonding and antibonding interaction of the orbitals.

The CASSCF calculations were performed with OpenMOLCAS,[S9] and DMRG-SCF calculations with the QCMaquis DMRG progam and its interface to OpenMOLCAS. The MPSSI approximation has been implemented in a in-house branch of QCMaquis.

References

(S1) Freitag, L.; González, L. The Role of Triplet States in the Photodissociation of a Platinum Azide Complex by a Density Matrix Renormalization Group Method. *J. Phys. Chem. Lett.* **2021**, *12*, 4876–4881.

(S2) Roos, B. O.; Lindh, R.; Malmqvist, P.-A.; Veryazov, V.; Widmark, P.-O. New Relativistic ANO Basis Sets for Transition Metal Atoms. *J. Phys. Chem. A* **2005**, *109*, 6575–6579.

(S3) Aquilante, F.; Pedersen, T. B.; Lindh, R.; Roos, B. O.; Sánchez de Merás, A.; Koch, H. Accurate Ab Initio Density Fitting for Multiconfigurational Self-Consistent Field Methods. *J. Chem. Phys.* **2008**, *129*, 024113.

(S4) Aquilante, F.; Gagliardi, L.; Pedersen, T. B.; Lindh, R. Atomic Cholesky Decompositions: A Route to Unbiased Auxiliary Basis Sets for Density Fitting Approximation with Tunable Accuracy and Efficiency. *J. Chem. Phys.* **2009**, *130*, 154107.

(S5) Pedersen, T.; Aquilante, F.; Lindh, R. Density Fitting with Auxiliary Basis Sets from Cholesky Decompositions. *Theor. Chem. Acc.* **2009**, *124*, 1–10.

(S6) Hess, B. A. Relativistic Electronic-Structure Calculations Employing a Two-Component No-Pair Formalism with External-Field Projection Operators. *Phys. Rev. A* **1986**, *33*, 3742–3748.

(S7) Wolf, A.; Reiher, M.; Hess, B. A. The Generalized Douglas–Kroll Transformation. *J. Chem. Phys.* **2002**, *117*, 9215–9226.

(S8) Reiher, M.; Wolf, A. Exact Decoupling of the Dirac Hamiltonian. II. The Generalized Douglas–Kroll–Hess Transformation up to Arbitrary Order. *J. Chem. Phys.* **2004**, *121*, 10945–10956.

(S9) Fdez. Galván, I.; Vacher, M.; Alavi, A.; Angeli, C.; Aquilante, F.; Autschbach, J.; Bao, J. J.; Bokarev, S. I.; Bogdanov, N. A.; Carlson, R. K.; Chibotaru, L. F.; Creutzberg, J.; Dattani, N.; Delcey, M. G.; Dong, S. S.; Dreuw, A.; Freitag, L.; Frutos, L. M.; Gagliardi, L.; Gendron, F.; Giussani, A.; González, L.; Grell, G.; Guo, M.; Hoyer, C. E.; Johansson, M.; Keller, S.; Knecht, S.; Kovačević, G.; Källman, E.; Li Manni, G.; Lundberg, M.; Ma, Y.; Mai, S.; Malhado, J. P.; Malmqvist, P. A.; Marquetand, P.; Mewes, S. A.; Norell, J.; Olivucci, M.; Oppel, M.; Phung, Q. M.; Pierloot, K.; Plasser, F.; Reiher, M.; Sand, A. M.; Schapiro, I.; Sharma, P.; Stein, C. J.; Sørensen, L. K.; Truhlar, D. G.; Ugandi, M.; Ungur, L.; Valentini, A.; Vancoillie, S.; Veryazov, V.; Weser, O.; Wesołowski, T. A.; Widmark, P.-O.; Wouters, S.; Zech, A.; Zobel, J. P.; Lindh, R. OpenMolcas: From Source Code to Insight. *J. Chem. Theory Comput.* **2019**, *15*, 5925–5964.
